# Psychological impact of COVID-19 on the Ecuadorian population: a comparative analysis 1 year after quarantine measures

**DOI:** 10.3389/fpsyg.2024.1383755

**Published:** 2024-09-05

**Authors:** Jorge Andrés Gallardo-Rumbea, María José Farfán Bajaña, Hans Mautong, Jorge Moncayo-Rizzo, Derly Andrade, Ivan Cherrez-Ojeda, Geovanny Alvarado-Villa

**Affiliations:** ^1^Universidad Espíritu Santo, Samborondon, Ecuador; ^2^Laboratorio de Ciencias Omicas, Facultad de Ciencias de la Salud, Universidad Espíritu Santo, Samborondón, Ecuador; ^3^Respiralab Research Group, Guayaquil, Ecuador

**Keywords:** COVID-19, stress, anxiety, depression, confinement, isolation

## Abstract

**Introduction:**

Social isolation during and after the COVID-19 pandemic has sparked interest in its psychological and neurobiological consequences. The pandemic has been associated with an increase in anxiety, depression, and stress, according to some cross-sectional studies. This study aims to analyze changes in the levels of anxiety, depression and stress by comparing the confinement phase to the post-confinement period in the Ecuadorian population.

**Methods:**

A longitudinal, comparative, prospective study was conducted using an online survey comprising two sections. The first section gathered demographic information, whereas the second section included the DASS-21 questionnaire. Ecuadorian participants who completed the survey during the initial data-collection period were included.

**Results:**

In total, 162 participants were included in the final analysis. The average age of the participants was 29.6 ± 11.7 years, and the majority were women (63.3%). In 2020, the median depression, anxiety, and stress scores were 6 (IQR 2–12), 6 (IQR 2–10), and 10 (IQR 6–16) respectively. In 2021, the median depression score was 8 (IQR 4–14), the median anxiety score was 8 (IQR 4–14.5), and the median stress and its interquartile range were 10 (IQR 6–18). The levels of depression, anxiety, and stress were significantly inversely correlated with age, number of children, self-reported general health, and self-reported mental health in both 2020 and 2021.

**Conclusion:**

Exercise, being a student, sex and having had COVID-19 examination may be predictors for the changes in the levels of psychological disorders. Implementing psychological strategies, such as cognitive behavioral therapy, and physiological interventions, like regular physical activity, early in the post-lockdown period could help mitigate the negative mental health impacts observed in the aftermath of the pandemic. These interventions can provide necessary support and coping mechanisms for those at higher risk, thereby improving overall mental health outcomes.

## 1 Introduction

Coronavirus, which swiftly spread across the globe, was declared a pandemic by WHO on March 11, 2020 (Cucinotta and Vanelli, 2020). Since then, there have been around 1 million confirmed cases and approximately 36 thousand deaths from COVID-19 (Henssler et al., 2021). Aside from the immediate health risks posed by the COVID-19 pandemic, widespread quarantine regulations have made psychological and neurobiological isolation a focal point of research (Henssler et al., 2021). Factors such as longer quarantine duration, financial loss, inadequate supplies, inadequate information, infection fears, frustration, boredom, and stigma may contribute significantly to the mental health impact of the COVID-19 pandemic (Lindert et al., 2021). A considerable number of cases of depressive syndrome and diminished overall well-being are linked to experiences of social isolation and loneliness worldwide (Holm-Hadulla et al., 2023). Numerous cross-sectional studies of the general population have documented elevated rates of anxiety, depression, and stress during the COVID-19 pandemic (Bueno-Notivol et al., 2021; Xiong et al., 2020; Amerio et al., 2021). In contrast, longitudinal studies conducted among have shown minimal to no alteration in anxiety or depression symptoms when compared to pre-pandemic levels leading to mounting concerns about mental health impacts (Penninx et al., 2022; O’Connor et al., 2021; Wang et al., 2020).

According to a study conducted in the United States, 43% of participants displayed heightened levels of loneliness, a condition correlated with depression and thoughts of suicide (Carvalho Aguiar Melo and de Sousa, 2020), particularly, among women, younger individuals, and those with lower educational attainment, the enforced social isolation resulting from pandemic-related restrictions contributed to feelings of depression and loneliness (Braun and Clarke, 2006; Seitz et al., 2021; Kuehner et al., 2020). Similar patterns were observed in studies from Spain, China, Italy, Austria, and Germany, among others, which showed an increase in anxiety and stress levels during peak confinement periods, as evidenced by the Anxiety and Stress Scale-21 Items (DASS-21) (González-Sanguino et al., 2021; Pieh et al., 2021; Zhang et al., 2021; Scandurra et al., 2023; Nisticò et al., 2021; Brailovskaia and Margraf, 2020). Contemporary reports have shown that long-term health affection and imposed deprivation of social contact, coupled with a feeling of lack of control, lead to a significant increase in mental health issues in the global population (Gobbi et al., 2020). A systematic review investigating the COVID-19 pandemic and mental health revealed that individuals with pre-existing psychiatric conditions experienced exacerbation of their symptoms during the lockdown period (Vindegaard and Benros, 2020). On the contrary, research indicated that approximately one-third of individuals with depressive syndromes experienced improvement following the easing of social restrictions (Holm-Hadulla et al., 2023). However, some research suggests that lockdowns still have negligible effects on students’ mental health despite the lifting of restrictions. Goldberg et al. reported that lockdowns and COVID-19 were associated with changes in depression and anxiety (Goldberg et al., 2022). Being female, young, unemployed, and having previous mental health problems were identified as sociodemographic factors associated with a higher risk of mental illness (Bonati et al., 2022). In addition, the persistence of symptoms of COVID or the appearance of new ones could be influenced by low educational level, economic difficulties, and sense of comfort (Camargo et al., 2023).

Lockdown measures have profoundly affected the daily life of the Ecuadorian population, impacting not only their health, but also economic, social, and psychological factors (Chocho-Orellana et al., 2022). Studies conducted in Ecuador have reported high levels of depression, anxiety, and stress, showing variations in prevalence rates during social isolation (Mautong et al., 2021). According to Chocho et al. 31.4, 39.7, and 22.8% of participants reported experiencing depression, anxiety, and stress, respectively (Chocho-Orellana et al., 2022). Compared to Mautong et al., 30.7% of respondents reported moderate to very severe anxiety, followed by depression (14.2%), and stress (17.7%) (Mautong et al., 2021). However, the impact of social isolation measures on the mental health of the Ecuadorian population remains unknown.

This study aims to analyze changes in scores of anxiety, depression, and stress comparing findings from the confinement phase to those from its aftermath. This will also allow us to analyze demographic conditions related to the three aforementioned psychological variables by comparing findings from 2020 to 2021. Thus, health authorities can bolster both mental and overall well-being and avoid risk factors that potentially trigger mental distress.

## 2 Methods

In Ecuador, the first case of COVID-19 was reported on February 29th, 2020, and a public health emergency was declared on March 11th, 2020. Subsequently, stringent measures were instituted commencing on March 17th, 2020. These measures included restrictions on mobility, the cessation of in-person activities in both professional and educational spheres, as well as the enforcement of lockdowns, a state of emergency, and curfews. These protocols remained in effect until January 3rd, 2021 when certain restrictions such as mobility constraints, curfews, and the state of emergency were eased. Nevertheless, telecommuting, online education, and the closure of land and sea borders persisted.

### 2.1 Study design and population

This longitudinal prospective analytic study was conducted over two periods. For data collection, an online survey, made using QuestionPro^®^, was applied to 38 questions. The first data collection was performed from April 22, 2020, to May 3, 2020, and data were published by Mautong et al. (2021). The second data collection was done from May 17, 2021, to October 2, 2021, coinciding with the easing of COVID-19 quarantine measures. The survey was sent to every participant who provided their contact information through social media channels (WhatsApp App, Email, or phone call) to avoid face-to-face interactions and maintain safe interactions during the pandemic. The message sent to the participants included a description of the purpose of the study alongside the URL. The survey, with an average completion time of 12 min, began with an introduction and electronic informed consent, followed by three modules focused on (1) Demographics and the DASS-21; (2) Exposure to COVID-19 and daily disruptions in life activities; and (3) overall health status.

The inclusion criteria were completion of the survey in the first data collection period and being in Ecuador at least 30 days before the survey was completed in the second data collection period. The exclusion criterion was the participants’ consciousness when filling out the survey. To identify which surveys were answered consciously and which were not, a pair of test questions was added. The test question consisted of choosing an answer dictated by the slogan. If the participant did not select the correct dictated answer, their response was considered biased and the participant was excluded from the study. To avoid duplicates, the QuestionPro^®^ IP-tracking feature verified the participants’ locations. A total of 626 participants were registered in the first data collection period (Mautong et al., 2021), of which 219 individuals completed the survey during the second data collection period. Further, 52 individuals’ data were excluded from the study based on the exclusion criteria, leaving 162 participants included in the final analysis.

### 2.2 Survey design

The survey consisted of several sections. The first section collected demographic information, such as sex, age, marital status, level of education, number of children, and profession. The second section included the questionnaire DASS-21, to evaluate the mental health status of socially isolated Ecuadorians during and after the COVID-19 quarantine period. For this study, a validated Spanish version of the DASS-21 was applied (Daza et al., 2002). The next section of the survey included questions on exposure, preventive measures, and daily life disruptions due to COVID-19. Additionally, this section registered information regarding the symptoms, diagnosis, and consequences of COVID-19. Finally, the last section included questions about habits such as sleep patterns, alcohol consumption, cigarette smoking, and drug use, as well as perceptions of general and mental health, and concerns about acquiring COVID-19. The latter three variables were assessed using a scale from 0 to 10, where 0 indicated no concerns about COVID-19/worst health and 10 indicated extreme concerns about COVID-19/best health.

#### 2.2.1 DASS-21 questionnaire

The DASS-21 is a 21 items scale divided into depression, anxiety, and stress subcategories, which are each measured on a 4-point (0–3) Likert-type scale (ranging from “did not apply to me at all” = 0 to “applied to me very much or most of the time” = 3). Participants were asked how much over the past month the statements in the DASS-21 had been applied to them. The depression subscale addresses dysphoria, hopelessness, devaluation of life, self-deprecation, lack of interest/involvement, anhedonia, and inertia. The anxiety subscale addressed autonomic arousal, skeletal muscle effects, situational anxiety, and the subjective experience of anxious affect. The stress subscale addresses difficulty relaxing, nervous arousal, and being easily upset/agitated, irritable/overreactive, and impatient. Scores for depression, anxiety, and stress were obtained by summing the individual items relevant to each scale and multiplying them by 2. The subscale scores ranged from 0 to 42, and the total scores ranged from 0 to 126. A score greater than 9 on the depression subscale, 7 on the anxiety subscale, and 14 on the stress subscale indicated positive results in these conditions. The reliability of the scale was assessed using Cronbach’s alpha coefficient, which indicated excellent reliability across both years (2020: α = 0.935 and 2021: α = 0.947). For 2020 subscales reliability was 0.834 for anxiety, 0.863 for depression, and 0.840 for stress. Whereas for 2021, anxiety subscale coefficient was 0.832, for depression was 0.911, and for stress was 0.849.

### 2.3 Statistical analysis

Quantitative variables were presented as means ± standard deviation (SD), median, and interquartile range (IQR), and qualitative variables were presented as percentages. Association analyses were performed using Wilcoxon’s test for comparison of the median scores, and correlations were performed using the Spearman test. Finally, linear regression was performed to assess the predictors of changes in depression, anxiety, and stress scores. The dependent variable was calculated by subtracting the 2020 variable score from the 2021 variable score, creating the variables delta anxiety, delta depression, and delta stress. The predictors included in the multiple linear regression analysis were determined by univariate regression analysis for each dependent variable, and all 2020 variables were initially analyzed. Finally, according to Mattes and Roheger (2020), mathematical reasons allow us to consider among the predictors the initial score of every dependent variable in linear regression analyses. However, this was not included in the interpretation, as the model may have diminished its reliability. All statistical analyses were performed using SPSS for Windows (version 23.0; SPSS Inc., Chicago, IL, United States).

### 2.4 Ethical considerations

This study was conducted in accordance with the Declaration of Helsinki and was approved by the Comité expedito de Ética of the Ministry of Health of Ecuador (Approval No. 024–2020). With the information collected in the survey, personal identification was not possible; as such, anonymity and protection of personal data were preserved.

## 3 Results

### 3.1 Sociodemographic characteristics of the study population

The baseline sociodemographic data for the 162 participants across 2020 and 2021 are shown in Table 1. In both years, participants had an average age of 29.6 ± 11.7 years, and consistently, the majority were women (63.3%). In 2020, 66.5% of the participants were identified as students. This attribute remained similar in 2021, where the majority (64.2%) were still students and the same percentage of singleness was maintained (73.5%).

**Table 1:** Baseline sociodemographic characteristics of 2020 and 2021.

Variables	2020 (*n* = 162)	2021 (*n* = 162)
Age (Mean ± SD)	26.96 ± 10.9	
Sex		
Male	59(36.4%)	
Female	103(63.6%)	
Marital status		
Single	123(73.5%)	119(73.5%)
Married	26(16%)	28(17.3%)
Widowed	1(0.6%)	1(0.6%)
Divorced	9(5.6%)	8(4.9%)
Cohabiting	3(1.9%)	6(3.7%)
Education level		
Primary education	0(0%)	2(1.2%)
Secondary education	85(52.5%)	77(47.5%)
Technician	1(0.6%)	7(4.3%)
University graduate	62(38.3%)	61(37.7%)
Post-graduate	14(8.6%)	15(9.3%)
Place of residence		
Province of Guayas	124(76.5%)	130(80.2%)
Guayaquil	65(40.1%)	67(41.33%)
Samborondón	38(23.4%)	39(24.06%)
Daule	15(9.2%)	20(12.33%)
Other provinces	6(3.7%)	4(2.46%)
Rest of the country	38(23.5%)	32(19.8%)
Active student status	107(66.5%)	104(64.2%)
Number of children (Median. IQR)	0 (0–5)	1 (1–6)
COVID-19 symptoms	28(17.3%)	13(8%)
COVID-19 diagnosis confirmed	10(6.2%)	20(12.3%)
Cigarettes consumption		
No	139 (85.8%)	129 (79.6%)
Yes	23 (14.2%)	33 (20.4%)
Less than last year	18 (11.1%)	16 (9.9%)
As same as last year	1 (0.6%)	8 (4.9%)
More than last year	4 (2.5%)	9 (5.6%)
Alcohol consumption		
No	88 (54.3%)	52 (32.1%)
Yes	74 (45.7%)	110 (67.9%)
Less than last year	69 (42.6%)	55 (34%)
As same as last year	4 (2.5%)	38 (23.5%)
More than last year	1 (0.6%)	17 (10.5%)
Sleep pattern		
Less than last year	39 (24.1%)	56 (34.6%)
As same as last year	47 (29%)	67 (41.4%)
More than last year	76 (46.9%)	39 (24.1%)

n, number; IQR, Interquartile range; COVID-19, Coronavirus disease 2019.

Most of the respondents (76.5%) in 2020 had already lived in the province of Guayas, but in 2021, there was a slight increase to 80.2% in that province. Approximately 17.3% of the participants experienced COVID-19 symptoms in 2020, while less than half (8%) reported experiencing COVID-19 symptoms in 2021. Only 6.2% of participants in 2020 had a confirmed diagnosis of COVID-19, compared to twice as many (12.3%) participants who had a confirmed diagnosis by a health professional in 2021.

In 2020, 45.7% of the population consumed alcohol and 14.2% consumed cigarettes during social confinement. In 2021, alcohol consumption increased by 22.2%, while cigarette consumption increased by 6.2%. The participants reported better sleep in 2020 (46.9%) than in 2021 (24.1%).

### 3.2 Categories of depression, anxiety, and stress

Table 2 shows the statistics of the DASS-21 for all participants in 2020 and 2021. Notably, there were significant differences in depression, anxiety, and stress levels between these 2 years. In 2020, the median depression score was 6 (IQR 2–12.5), with approximately 24.7% of participants reporting moderate to very severe depression levels. The median anxiety score was 6 (IQR 2–10.5), and 33.3% reported moderate to very severe anxiety levels. The stress score had a median of 10 (IQR 6–16), which was higher than the anxiety and depression scores. Despite this, the proportion of participants with moderate to very severe stress levels was 15.5%.

**Table 2:** Categories of depression, anxiety, and stress.

Variables		2020			2021			*p*-value*
		*n* = 162	Percentage	Median (IQR)	*n* = 162	Percentage	Median (IQR)	
Anxiety	Normal	92	56.8%	6 (2–10.5)	71	43.8%	8 (4–14.5)	<0.001
	Mild	16	9.9%		14	8.6%		
	Moderate	37	22.8%		37	22.8%		
	Severe	5	3.1%		16	9.9%		
	Extremely severe	12	7.4%		24	14.8%		
Depression	Normal	96	59.3%	6 (2–12.5)	94	58.0%	8 (4–14)	0.215
	Mild	26	16.0%		20	12.3%		
	Moderate	24	14.8%		25	15.4%		
	Severe	5	3.1%		12	7.4%		
	Extremely severe	11	6.8%		11	6.8%		
Stress	Normal	118	72.8%	10 (6–16)	106	65.4%	10 (6–18)	0.056
	Mild	19	11.7%		19	11.7%		
	Moderate	11	6.8%		19	11.7%		
	Severe	11	6.8%		14	8.6%		
	Extremely severe	3	1.9%		4	2.5%		

* *p*-value calculated with Wilcoxon test (for median scores).

**Figure 1:**
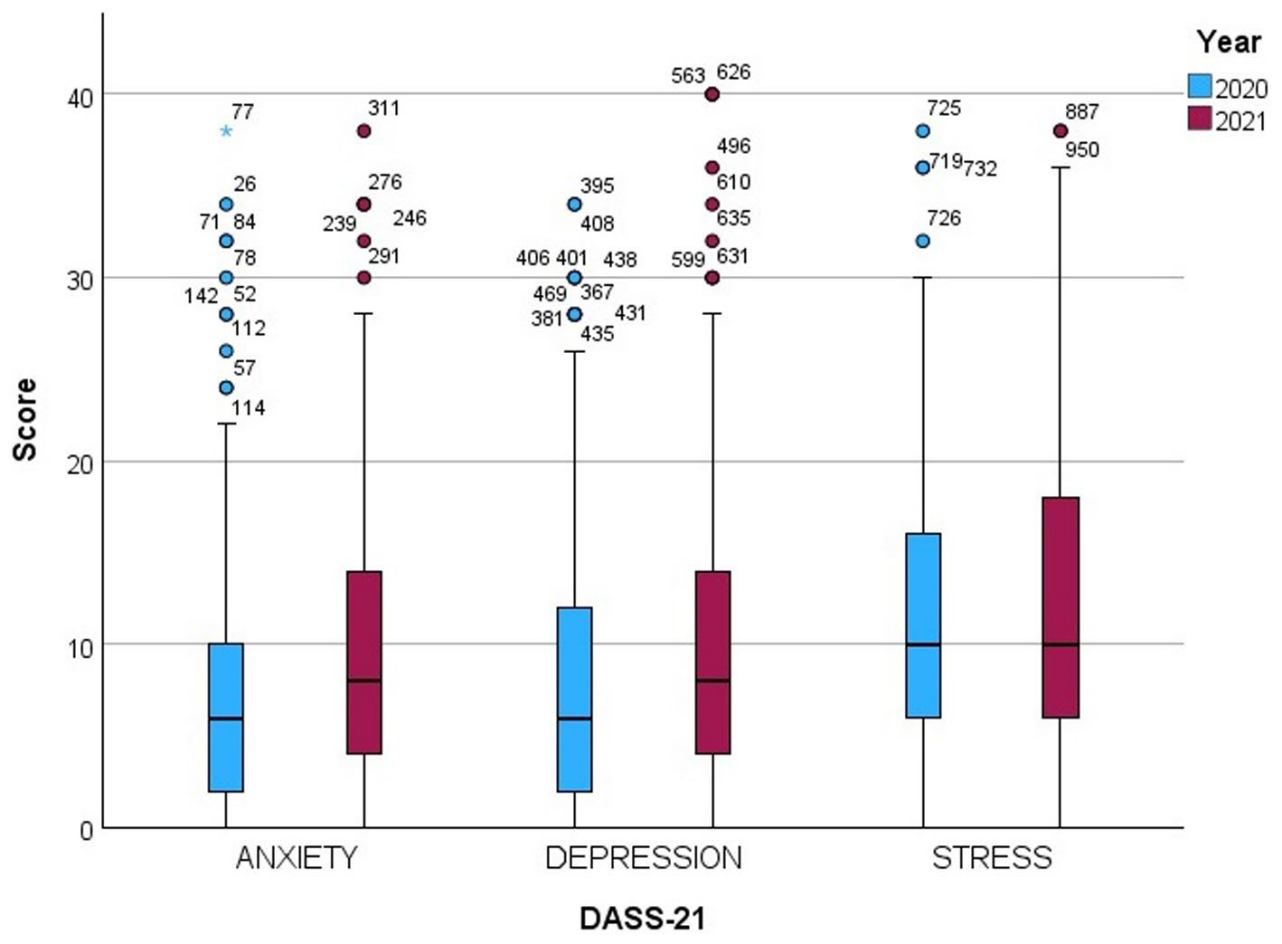
Comparison between score levels of depression, anxiety, stress in 2020 and 2021.

In contrast, the 2021 results showed an increase in the median depression score to 8 (IQR 4–14), and approximately 29.6% of the participants presented moderate to very severe levels of depression. The median anxiety score was 8 (IQR 4–14.5), while moderate to very severe anxiety levels were approximately 47.5%. The median stress and interquartile range were 10 (IQR 6–18), and 22.8% yet reported moderate to very severe stress levels. Figure 1 presents the levels of anxiety, depression, and stress in both years. Moreover, Supplementary Table S1 shows the median scores for anxiety, depression, and stress levels in the years 2020 and 2021, according to the categories of each variable.

### 3.3 Association analyses

Associations between qualitative variables and anxiety (Supplementary Table S2), depression (Supplementary Table S3), and stress (Supplementary Table S4) scores were determined with U-Mann Witney and Kruskal-Wallis tests. A significant association was identified between sex and the anxiety, depression, and stress level scores in both years, indicating that men presented less anxiety, depression, and stress levels. Habits such as alcohol consumption, cigarette consumption, and exercise were also analyzed; however, the results showed that none of these were significantly associated with the scores. Moreover, COVID-19 symptoms, examinations, and diagnoses in 2020 were analyzed. Only the COVID-19 examination in 2020 was significantly associated with the 2021 stress score, and a similar non-significant trend was found between this variable and the 2021 anxiety and depression scores.

Correlations among quantitative variables were analyzed with Spearman’s correlation test. As shown in Table 3, levels of depression, anxiety, and stress had significant inverse correlations with age, number of children, self-reported general health, and self-reported mental health in both 2020 and 2021. On the other hand, participants’ level of concern about contracting COVID-19 had significant positive direct correlations with levels of depression, anxiety, and stress in 2020 and 2021.

**Table 3:** Associations between continuous variables and depression, anxiety, and stress scores in 2020 and 2021.

2020 variables	Depression	*p*-value	Anxiety	*p*-value	Stress	*p*-value^a^
Age	−0.411**	<0.001	−0.247**	0.001	−0.321**	<0.001
Number of children	−0.330**	<0.001	−0.187**	0.009	−0.196**	0.006
Subjective perception of general health	−0.257**	<0.001	−0.252**	0.001	−0.290**	<0.001
Subjective perception of mental health	−0.609**	<0.001	−0.428**	<0.001	−0.515**	<0.001
Worry about acquiring COVID-19	0.156*	0.024	0.230**	0.002	0.214**	0.003
2021 variables	Depression	p-value	Anxiety	p-value	Stress	p-value
Age	−0.204*	0.009	−0.251**	0.001	−0.239*	0.002
Number of children	−0.235*	0.003	−0.207*	0.008	−0.199*	0.011
Subjective perception of general health	−0.281**	<0.001	−0.325**	<0.001	−0.311**	<0.001
Subjective perception of mental health	−0.681**	<0.001	−0.605**	<0.001	−0.695**	<0.001
Worry about acquiring COVID-19	0.104	0.190	0.134	0.090	0.139	0.077

a *p*-values were calculated using a Spearman correlation test. *p = <0.05; **p = <.001.

### 3.4 Linear regression analysis

Linear regression analysis was performed to determine the predictors of changes in depression, anxiety, and stress scales. The dependent variable was calculated by subtracting the 2020 variable score from the 2021 variable score. The results of these new variables are interpreted as follows: a positive change in the score indicates increased levels of anxiety, depression, or stress for 2021 in relation to 2020; in contrast, negative values indicate a decrease in anxiety, depression, or stress levels in 2021.

Prior to the multivariate regression analysis, a univariate regression analysis was conducted with all the 2020 variables to identify potential predictors. Variables that significantly predicted changes in depression, anxiety, and stress levels in the univariate analysis (Coefficients are shown in Table 4, non-significant predictors were not included in the table) were included in the multivariable analysis.

**Table 4:** Univariate and multivariate analysis for variables’ coefficients that predict changes in depression.

	Univariate analysis				Multivariate analysis				
Predictors	Coefficient b	CI (95%)		*p*-value	Coefficient b	Beta coefficient	CI (95%)		*p*-value
Dependent variable: change in depression									
(2020) COVID-19 examination (yes)	−7.556	−12.383	−2.729	0.002	−5.402	−0.152	−10.315	−0.490	0.031
(2020) Exercise (same as a year ago)	2.726	−0.422	5.874	0.089	2.790	0.131	−0.290	5.870	0.075
(2020) Exercise (more than a year ago)	5.299	2.496	8.101	<0.001	4.466	0.238	1.699	7.234	0.002
(2020) Student (yes)	3.537	0.844	6.229	0.010	3.195	0.176	0.593	5.797	0.016
(2020) depression score					−0.409	−0.389	−0.561	−0.258	<0.001
Self-reported mental health (2020)	−1.014	−1.745	−0.282	0.007					
(2020) COVID-19 symptoms (yes)	3.493	0.235	6.751	0.036					
Dependent variable: change in anxiety									
Sex (female)	2.595	0.416	4.774	0.020	2.220	0.150	0.189	4.250	0.032
(2020) Student (yes)	3.756	1.570	5.942	<0.001	2.689	1.074	0.567	4.810	0.013
Self-reported mental health	−1.191	−1.716	−0.667	<0.001	−1.026	−0.299	−1.543	−0.510	<0.001
(2020) Anxiety score					−0.497	−0.529	−0.637	−0.356	<0.001
Age	−0.116	−0.211	−0.020	0.018					
(2020) COVID-19 examination (yes)	−4.989	−9.090	−0.888	0.017					
Self-reported general health	−1.091	−1.835	−0.347	0.004					
Worry about acquiring COVID-19	−0.451	−0.878	−0.024	0.038					
Dependent variable: change in stress									
(2020) Student (yes)	3.692	1.187	6.197	0.004	2.976	0.171	0.565	5.386	0.016
(2020) COVID-19 examination (yes)	−7	−11.541	−2.459	0.003	−6.153	−0.181	−10.674	−1.631	0.008
Self-reported mental health	−1.201	−1.836	−0.567	<0.001	−1.032	−0.261	−1.642	−0.412	0.001
(2020) Stress score					−0.602	−0.586	−0.762	−0.441	<0.001
Sex (female)	2.922	0.482	5.363	0.019					

For the change in depression scores, the variables included were self-reported mental health, COVID-19 symptoms and examinations, exercise, and being a student. After the analysis, the statistically significant (*p* < 0.001) model for change in depression included COVID-19 examination, exercise, and being a student, with a Durbin-Watson test score of 2.225, indicating no autocorrelation. The mathematical model for change in depression was $\Delta \mathrm{Depression}=1.039-5.402x_{1}+2.790x_{2}+4.467x_{3}+3.195x_{4}-0,409x_{5}$ , which explains the 24.8% of the variance. The model shows that having been a student and exercising more in 2021 than during the previous year predicted a positive change in the level of depression from 2020 to 2021. On the other hand, having undergone a COVID-19 test predicts a negative change in depression levels, indicating a greater decrease in it.

For changes in anxiety scores, the variables included were COVID-19 examinations, sex, age, being a student, worry about acquiring COVID-19, self-reported general health, and self-reported mental health. Due to multicollinearity (VIF for age: 2.490 and VIF for student: 2.258), the variable “age” was withdrawn from the regression model. After the analysis, the statistically significant (*p* < 0.001) model for changes in anxiety included sex, being a student, and self-reported mental health, with a Durbin-Watson test of 1.996, indicating no autocorrelation. The mathematical model for change in anxiety was $\Delta \mathrm{Anxiety}=11.453+2.220x_{1}+2.689x_{2}-1.026x_{3}-0.497x_{4}$ , which explains the 25.6% of the variance. The model shows that being female and being a student predict a positive change in the level of anxiety, indicating an increase in levels or a lesser decrease in it. Meanwhile, better self-reported mental health predicts a negative change, indicating a greater decrease in anxiety levels.

Finally, for changes in stress scores, the variables included were being a student, COVID-19 examination, self-reported mental health, and sex. After the analysis, the statistically significant (p < 0.001) model for change in stress included being a student, COVID-19 examination, and self-reported mental health, with a Durbin-Watson test of 2.052, indicating no autocorrelation. The mathematical model for change in stress was $\Delta \mathrm{Stress}=15.262+2.976x_{1}-6.153x_{2}-1.032x_{3}-0.602x_{4}$ , which explains 27.5% of the variance. The model shows that having been a student predicts a positive change in stress levels; meanwhile, having undergone a COVID-19 test and reporting better mental health predict a negative change, indicating a greater decrease in stress levels.

## 4 Discussion

In our study, 63.3% of patients were female, similar to the findings of Kupcova et al., who reported that 62% of patients were female (Kupcova et al., 2023). In the same study, 76.6% were university students, while in our study 66.5% were identified as students in both 2020 and 2021 (Kupcova et al., 2023). Various studies have identified additional factors linked to the decline in mental health during the COVID-19 pandemic, including heightened cigarette and alcohol consumption and sleep-related issues (Ramalho, 2020; Clay and Parker, 2020). The reported alcohol consumption increased by 22.2% in our study, which is consistent with the explanation that throughout history, there has been a connection between economic crises and respiratory epidemics leading to higher levels of alcohol consumption (Gonçalves et al., 2020). In our study, cigarette consumption increased by 6.2%. According to a study in Brazil during the Covid-19 pandemic, 20 and 30% of individuals reported an increase in alcohol consumption and cigarette smoking, respectively (Schäfer et al., 2022). Furthermore, the participants in our study reported a 22.8% decrease in sleep from 2020 to 2021. This finding is consistent with studies that also showed increased cigarette consumption and sleep problems in the majority of the young adult population in 2021 due to the pandemic (Martínez-Cao et al., 2021; Islam et al., 2020; Alimoradi et al., 2021).

In our study, which was conducted 1 year after lockdown measures were implemented, approximately 29.6, 47.5, and 22.8% of the participants presented moderate to very severe levels of depression, anxiety, and stress, respectively. A prior study of Ecuadorian participants conducted during 2020 in the wake of the pandemic showed that 17.7% of those surveyed experienced depression, 30.7% had moderate-to-severe anxiety, and 14.2% experienced stress (Mautong et al., 2021). Several hypotheses have been raised regarding whether social isolation or the resulting measures in the aftermath of the pandemic have led to deteriorated mental health and adverse effects on individuals and their environment. A longitudinal study in the UK suggested that mental health improved during the COVID-19 pandemic due to reduced working hours and government subsidies (Fancourt et al., 2021). Similarly, researchers in Colombia found that many participants who began quarantine with good mental health also improved their mental health over the course of quarantine, especially in terms of anxiety, psychological resilience, and perceived social support (Yu et al., 2023). Despite this, evidence suggests that mental health deteriorated in the aftermath of the COVID-19 lockdowns accompanied by reduced life satisfaction and loneliness (Grimes, 2022).

Our study revealed significantly lower levels of depression, anxiety, and stress among men than women in both years. In addition, from the results of the regression model, female sex was a predictor of a positive change in anxiety scores. This indicates that being female may lead to increased anxiety scores or may lead to a lesser decrease in scores compared to men. Some studies suggest that males are associated with reduced odds of experiencing stress (Harries et al., 2021; Zamorano González et al., 2021; Bonilla-Sierra et al., 2021). In addition, a sociocultural perspective may explain these findings, which show that women in societies traditionally bear more domestic burdens than men, magnifying gender inequalities and domestic violence, especially when they are socially isolated (Wang et al., 2020; Tee et al., 2020; Alharbi et al., 2021; Vancea and Apostol, 2021; Picó-Pérez et al., 2021; Gopal et al., 2020; Chiaramonte et al., 2022; Del Río-Casanova et al., 2021). A Romanian study reported there were no significant changes in levels of depression, anxiety, or stress during the COVID-19 crisis, however, one in three women exhibited elevated levels of at least one of these symptoms (Vancea and Apostol, 2021).

We found a significant inverse correlation between the number of children and the levels of depression, anxiety, and stress. In the US, about half of the parents of children under 18 years of age reported high levels of stress during the pandemic (Pudpong et al., 2023). In Latin America, the prevalence of anxiety and depression among parents during the pandemic was 22.1 and 26.6%, respectively (Ben Brik et al., 2022). According to a study conducted in Norway, the number of children may be associated with parental stress (Johnson et al., 2022). In addition, several studies that used the DASS-21 identified having children as a protective factor against changes in the mental health status of people who witnessed the period of social isolation (Wang et al., 2020; Tee et al., 2020; Del Río-Casanova et al., 2021; Wang et al., 2021). In contrast, other studies state an increase in psychological distress compared to previous years among people living with children (Picó-Pérez et al., 2021; Roma et al., 2020). Although the results mentioned earlier, there is also evidence that having a dysfunctional family could increase the incidence rates of depression and anxiety during the stay-home period of the pandemic (Liu et al., 2023).

A systematic review of 146,139 subjects from more than 14 countries reported anxiety, depression, and stress levels of 35.9, 29.7, and 12.5%, respectively (Rabiu Abubakar et al., 2022). Evidence suggests that epidemics or natural disasters increase the long-term levels of depression in populations (Rodríguez-Hidalgo et al., 2020). This could easily be explained by the drastic labor and economic change that the population faced after confinement, a period of economic recession, unemployment, teleworking, and the restriction of mobilization, which limited the possibility of financial recovery (Tee et al., 2020; Amagua et al., 2022). On the other hand, the return of children and young people to face-to-face classes could also explain the high levels of stress reported, since this involves a high economic expense, reorganization of the usual schedule, and adaptation of sanitary measures in face-to-face education, among others (Ozamiz-Etxebarria et al., 2021; O’Byrne et al., 2021; Bashirian et al., 2021). Bashirian’s study in Iran shows us an example where 90% of the participants were stressed and worried about the social distancing plan and reopening of schools during the COVID-19 pandemic (Bashirian et al., 2021). Other investigations reported loneliness as an important predictor of high levels of anxiety and depression as a result of social distancing, confinement, and mobility restrictions imposed to stop the pandemic (Tee et al., 2020).

A significant association was found between examination COVID of 2020 and stress levels in 2021, which suggests that stress levels were lower in those who had a COVID-19 examination. Moreover, a similar trend was found for 2021 levels of depression. On the other hand, presenting symptoms of COVID-19 and/or its diagnosis confirmed by a professional were not associated with significantly higher levels of anxiety, depression, or stress in 2020 or 2021. Literature shows that having a family member diagnosed with COVID-19 was associated with more severe levels of depression and anxiety (Mautong et al., 2021). One explanation for this may be that the relatives are more concerned than the patient about his illness (Tacchini-Jacquier et al., 2023). A recent meta-analysis concluded that family support factors had an inverse effect on depression (Shao et al., 2024).

In our regression, age wasn’t a predictor for negative change in anxiety, in contrast to study by Fenollar-Cortés’ in which they conducted a 3-month longitudinal pandemic study using the DASS-21 and it showed participants had higher anxiety, stress, and depressive symptomatology (Fenollar-Cortés et al., 2021). Likewise, other studies have reported lower depression and anxiety in older adults than in 18–30-year-old individuals (Lind et al., 2021; Zhu and Upenieks, 2022). Levels of religiosity, secure attachment to God, and better strategies for coping with negative emotions were proposed as reasons why these findings could occur (Zhu and Upenieks, 2022). However, in a study from the UK, Gaggero et al. reported that levels of depression worsened in older adults, especially in those who lost their jobs as a consequence of the pandemic, retired individuals, insufficient social support, and women (Gaggero et al., 2022).

Student status was associated with higher scores in depression, anxiety, and stress in 2021 compared to 2020. Plakhotnik et al. illustrate how university support during pandemic time enhance the student’s perceptions and emotional states, including stress, and life satisfaction (Plakhotnik et al., 2021).

COVID-19 and the measures implemented to curb it have had an immediate negative impact on mental health, as reported by Mautong et al. (2021) in a previous study in 2020. One year after those findings, our results indicate a deterioration in the psychological profile in 2021, which highlights the relevance of this longitudinal follow-up study regarding changes in anxiety, depression, and stress levels. The benefit of establishing a temporal analysis within this longitudinal study is that it provides insights into the long-term mental health challenges linked to confinement. The applicability of this information is crucial for the successful implementation of measures to support individuals affected by such challenges. Utilizing the available data effectively, healthcare professionals and support organizations can tailor interventions to address specific needs and mitigate adverse effects. This approach enhances the efficiency and effectiveness of support systems, ensuring that the allocated resources meet the evolving needs of affected individuals. However, its limitations must be recognized. The use of an online self-report questionnaire might have introduced several sources of bias (self-report bias, selection bias, and sampling bias). Consequently, some categories of people were underrepresented (for example, married adults, adults with children, residents of other provinces, etc.). No clinical psychological or psychiatric diagnosis was made, which prevented the investigator from determining whether the nature of the symptoms was psychological or psychiatric. Therefore, the results should be viewed with caution, and additional studies are required to assess whether these results can be replicated in other populations using different sampling methods. The reduction of the sample size from 646 to 162 participants did not allow a precise comparison, which gave us non-generalizable results. However, the findings of this study provide valuable information about the mental health of a Latin American country during and 1 year after social isolation. That is, it contributes to information about long-term mental disorders associated with confinement and data with limited availability.

## 5 Conclusion

Exercise and being a student were positive predictors of changes in depression levels. Sex and being a student were a positive predictors for changes in anxiety levels. COVID examination was a negative predictor for stress levels. Early implementation of psychological strategies and physiological interventions could help attenuate mental health repercussions in the aftermath of the pandemic.

## Data Availability

The raw data supporting the conclusions of this article will be made available by the authors, without undue reservation.
