# Supplementary material for: Psychological impact of COVID-19 on the Ecuadorian population: a comparative analysis 1 year after quarantine measures
Source: Front Psychol. 2024 Sep 5;15:1383755. doi: 10.3389/fpsyg.2024.1383755 (PMC11412256; doi:10.3389/fpsyg.2024.1383755)
Supplement: Supplementary file 1 [file Table_1.docx]

Supplementary Material

Psychological Impact of COVID-19 on the Ecuadorian population: A comparative analysis one year after quarantine measures

[Jorge Andrés Gallardo-Rumbea](https://pubmed.ncbi.nlm.nih.gov/?term=Gallardo-Rumbea+JA&cauthor_id=30674297)^1^ , [María José Farfán Bajaña](https://pubmed.ncbi.nlm.nih.gov/?term=Farf%C3%A1n+Baja%C3%B1a+MJ&cauthor_id=34812287)^1^, Hans Mautong^1^, Jorge Moncayo-Rizzo^1^, Derly Andrade^2^, Ivan Cherrez-Ojeda^1,3^, Geovanny Alvarado-Villa^1^

*** Correspondence:** Geovanny Alvarado-Villa [galvarado@uees.edu.ec](mailto:galvarado@uees.edu.ec)

# Supplementary Data

**Supplementary appendix**

***Table 1S. Median scores of anxiety, depression and stress for 2020 and 2021 according to each variable categories.***

|  | | **Anxiety score 2020** | | **Anxiety score 2021** | |
| --- | --- | --- | --- | --- | --- |
|  |  | **Count** | **Median** | **Count** | **Median** |
| **Anxiety categories** | Normal | 92 | 2 | 71 | 2 |
|  | Mild | 16 | 8 | 14 | 8 |
|  | Moderate | 37 | 12 | 37 | 12 |
|  | Severe | 5 | 18 | 16 | 16 |
|  | Extremely Severe | 12 | 28 | 24 | 24 |
|  | | **Depression score 2020** | | **Depression score 2021** | |
| **Depression categories** | Normal | 96 | 4 | 94 | 4 |
|  | Mild | 26 | 10 | 20 | 10 |
|  | Moderate | 24 | 16 | 25 | 16 |
|  | Severe | 5 | 22 | 12 | 23 |
|  | Extremely Severe | 11 | 30 | 11 | 32 |
|  | | **Stress score 2020** | | **Stress score 2021** | |
| **Stress categories** | Normal | 118 | 8 | 106 | 8 |
|  | Mild | 19 | 16 | 19 | 16 |
|  | Moderate | 11 | 20 | 19 | 22 |
|  | Severe | 11 | 26 | 14 | 30 |
|  | Extremely Severe | 3 | 36 | 4 | 37 |

***Table 2S. Association between sociodemographic variables and anxiety in 2020 and 2021.***

|  | | Anxiety 2020 | | p value | Anxiety 2021 | | p value |  |
| --- | --- | --- | --- | --- | --- | --- | --- | --- |
|  |  | Mean | SD |  | Mean | SD |  |  |
| Sex | Male | 5.93 | 6.17 | 0.035* | 7.25 | 6.77 | 0.001* |  |
|  | Female | 8.68 | 8.19 |  | 11.59 | 8.87 |  |  |
| Alcohol 2020 | I do not consume | 7.52 | 6.81 | 0.900 | 9.61 | 7.73 | 0.415 |  |
|  | Less than a year ago | 7.91 | 8.54 |  | 10.12 | 8.66 |  |  |
|  | As same as a year ago | 7.50 | 11.12 |  | 11.00 | 16.04 |  |  |
|  | More than a year ago | 4.00 |  |  | 28.00 |  |  |  |
| Cigarettes 2020 | I do not consume | 8.17 | 7.88 | 0.069 | 10.55 | 8.74 | 0.151 |  |
|  | Less than a year ago | 5.56 | 5.07 |  | 7.78 | 5.26 |  |  |
|  | As same as a year ago | 0.00 |  |  | 2.00 |  |  |  |
|  | More than a year ago | 2.00 | 2.31 |  | 3.50 | 2.52 |  |  |
| Exercise 2020 | Less than a year ago | 8,02 | 8,32 | 0,388 | 10.17 | 8.49 | 0,595 |  |
|  | As same as a year ago | 8,91 | 8,32 |  | 10.85 | 8.52 |  |  |
|  | More than a year ago | 6,25 | 5,51 |  | 9.17 | 8.30 |  |  |
| COVID-19 Symptoms 2020 | Yes | 9.93 | 9.94 | 0.269 | 10.29 | 10.45 | 0.612 |  |
|  | No | 7.21 | 6.99 |  | 9.96 | 7.96 |  |  |
| COVID-19 Examination 2020 | No | 7.67 | 7.79 | 0.421 | 10.34 | 8.54 | 0.061 |  |
|  | Yes | 7.82 | 4.94 |  | 5.45 | 4.39 |  |  |
| COVID-19 Diagnosis 2020 | Yes | 7.60 | 7.47 | 0.994 | 10.00 | 11.39 | 0.583 |  |
|  | No | 7.68 | 7.65 |  | 10.01 | 8.23 |  |  |
| *p value <0.05 calculated by U-Mann Witney test or Kruskal-Wallis test | | | | | | | |  |

***Table 3S. Association between sociodemographic variables and depression in 2020 and 2021.***

|  | | Depression 2020 | | p value | Depression 2021 | | p value |
| --- | --- | --- | --- | --- | --- | --- | --- |
|  |  | Mean | SD |  | Mean | SD |  |
| Sex | Male | 7.46 | 7.30 | 0.031* | 7.76 | 8.36 | 0.007* |
|  | Female | 10.08 | 8.55 |  | 11.36 | 9.56 |  |
| Alcohol 2020 | I do not consume | 8.76 | 7.50 | 0.711 | 10.41 | 8.85 | 0.246 |
|  | Less than a year ago | 9.51 | 8.91 |  | 9.10 | 9.26 |  |
|  | As same as a year ago | 11.50 | 12.37 |  | 15.00 | 17.01 |  |
|  | More than a year ago | 2.00 |  |  | 26.00 |  |  |
| Cigarettes 2020 | I do not consume | 9.38 | 8.19 | 0.220 | 10.45 | 9.39 | 0.263 |
|  | Less than a year ago | 8.78 | 8.90 |  | 8.67 | 9.13 |  |
|  | As same as a year ago | 0.00 |  |  | 0.00 |  |  |
|  | More than a year ago | 4.00 | 2.31 |  | 5.00 | 2.58 |  |
| Exercise 2020 | Less than a year ago | 10,35 | 8,63 | 0,094 | 8.69 | 7.94 | 0,171 |
|  | As same as a year ago | 8,67 | 7,48 |  | 10.36 | 10.71 |  |
|  | More than a year ago | 7,38 | 7,7 |  | 12.13 | 10.09 |  |
| COVID-19 Symptoms 2020 | Yes | 11.00 | 11.31 | 0.880 | 8.29 | 9.52 | 0.105 |
|  | No | 8.73 | 7.38 |  | 10.42 | 9.22 |  |
| COVID-19 Examination 2020 | No | 8.87 | 7.98 | 0.307 | 10.41 | 10.41 | 0.050 |
|  | Yes | 12.55 | 10.59 |  | 5.09 | 5.09 |  |
| COVID-19 Diagnosis 2020 | Yes | 11.20 | 12.48 | 0.875 | 8.40 | 12.29 | 0.192 |
|  | No | 8.99 | 7.87 |  | 10.16 | 9.09 |  |
| *p value <0.05 calculated by U-Mann Witney test or Kruskal-Wallis test | | | | | | | |

***Table 4S. Association between sociodemographic variables and stress in 2020 and 2021.***

|  | | Stress 2020 | | p value | Stress 2021 | | p |
| --- | --- | --- | --- | --- | --- | --- | --- |
|  |  | Mean | SD |  | Mean | SD | value |
| Sex | Male | 9.66 | 6.53 | 0.036* | 10.03 | 7.86 | <0.001* |
|  | Female | 12.66 | 8.59 |  | 14.58 | 8.95 |  |
| Alcohol 2020 | I do not consume | 10.74 | 7.50 | 0.623 | 13.03 | 8.22 | 0.299 |
|  | Less than a year ago | 12.72 | 8.66 |  | 12.32 | 9.12 |  |
|  | As same as a year ago | 11.50 | 8.70 |  | 16.50 | 14.27 |  |
|  | More than a year ago | 8.00 |  |  | 32.00 |  |  |
| Cigarettes 2020 | I do not consume | 11.55 | 8.15 | 0.485 | 13.53 | 9.06 | 0.093 |
|  | Less than a year ago | 12.56 | 7.79 |  | 9.56 | 6.64 |  |
|  | As same as a year ago | 2.00 |  |  | 0.00 |  |  |
|  | More than a year ago | 10.00 | 2.83 |  | 10.50 | 1.00 |  |
| Exercise 2020 | Less than a year ago | 12,32 | 8,48 | 0,461 | 12.20 | 8.81 | 0,432 |
|  | As same as a year ago | 11,52 | 7,86 |  | 13.94 | 9.12 |  |
|  | More than a year ago | 10,33 | 7,28 |  | 13.46 | 8.71 |  |
| COVID-19 Symptoms 2020 | Yes | 12.64 | 9.65 | 0.727 | 12.57 | 10.25 | 0.429 |
|  | No | 11.34 | 7.65 |  | 13.00 | 8.53 |  |
| COVID-19 Examination 2020 | No | 11.55 | 8.19 | 0.595 | 13.39 | 8.85 | 0.004* |
|  | Yes | 11.82 | 5.17 |  | 6.55 | 5.59 |  |
| COVID-19 Diagnosis 2020 | Yes | 11.20 | 9.53 | 0.727 | 11.40 | 11.51 | 0.234 |
|  | No | 11.59 | 7.94 |  | 13.03 | 8.66 |  |
| *p value <0.05 calculated by U-Mann Witney test or Kruskal-Wallis test | | | | | | | |
